# Case Report: Successful application of endoscopic biliary accessories in a patient with complete esophageal stricture

**DOI:** 10.3389/fmed.2025.1540103

**Published:** 2025-07-02

**Authors:** Junjun Yan, Qiangming Liao, Bin Ding

**Affiliations:** ^1^Jiujiang City Key Laboratory of Cell Therapy, Department of Gastroenterology, The First People’s Hospital of Jiujiang City, Jiujiang, China; ^2^Jiangxi Provincial Key Laboratory of Digestive Diseases, Jiangxi Clinical Research Center for Gastroenterology, Department of Gastroenterology, Digestive Disease Hospital, The First Affiliated Hospital, Jiangxi Medical College, Nanchang University, Nanchang, China; ^3^Jiujiang City Key Laboratory of Cell Therapy, Department of Gastrointestinal Surgery, The First People’s Hospital of Jiujiang City, Jiujiang, China

**Keywords:** anastomosis stenosis, biliary accessories, dilation, esophagectomy, case report

## Abstract

The endoscopic treatment of complete esophageal stricture presents a huge challenge. In instances where the esophageal lumen is nearly obliterated, preventing the passage of a dilating guide wire, conventional endoscopic bougie dilation is often difficult to perform. We report a case of patients with nearly complete esophageal stricture due to esophageal cancer surgery, leading to severe dysphagia and weight loss. Our strategy began with the achievement of anterograde esophageal access, facilitated by a biliary intubation method and aided by a 0.35-inch guidewire. Subsequently, we sequentially applied biliary dilators and balloon dilators, progressing along the guidewire to incrementally dilate the esophageal lumen up to the site of complete stenosis. The stenosis was successfully penetrated and dilated, and eventually the patient’s dysphagia was completely relieved. This case underscores the efficacy of the novel endoscopic treatment for biliary appendices in patients with refractory esophageal anastomotic stricture, offering a new therapeutic approach for clinical management.

## Introduction

Esophageal cancer postoperative anastomotic stricture is a prevalent complication that significantly impacts patients’ quality of life and prognosis. This condition can lead to debilitating symptoms such as dysphagia, regurgitation, and weight loss, adversely affecting the overall well-being of patients. Research indicates that esophageal strictures can be effectively treated using various endoscopic techniques (1). However, traditional management approaches may not always yield satisfactory outcomes, particularly in refractory cases. In this case, we report a successful pre-dilation of a refractory esophagogastric anastomotic stricture (EAS) using an endoscopic biliary guidewire and biliary dilation bougie, facilitating subsequent endoscopic treatment.

## Case report

A 66-year-old man came to the hospital complaining of repeated and worsening dysphagia over the past two years. The patient had a medical history of hypertension and atrial fibrillation. In early 2005, he developed a primary squamous cell carcinoma in the esophageal, and therefore underwent partial esophagectomy with mediastinal lymph node dissection and cervical gastro-esophagogastrostomy. Following the surgical procedure, the patient experienced dysphagia. An upper gastrointestinal endoscopy revealed incomplete stenosis at the anastomosis site. The initial treatment focuses on dilating the anastomosis by using wire-guided rigid polyethylene dilators such as Savary-Gilliard to maintain the patient’s liquid diet. However, the patient’s dysphagia worsened due to the development of esophageal stricture post-radiotherapy for esophageal cancer. Prior to our intervention, esophageal dilatation and esophageal stent placement were attempted several times to relieve esophageal obstruction; however, these efforts were unsuccessful due to scar shrinkage and stent migration.

**Figure 1:**
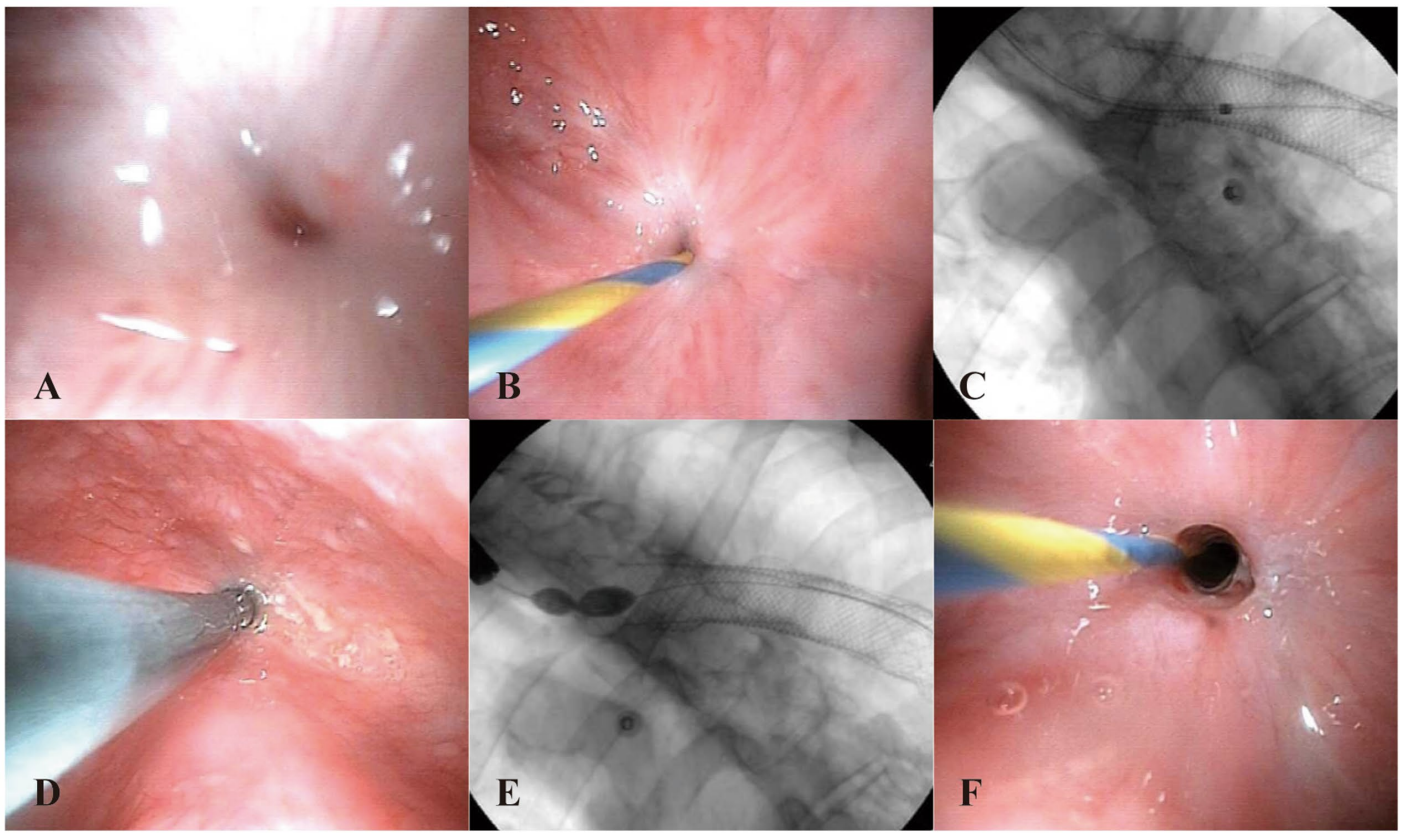
**(A)** Endoscopic view of the esophageal anastomosis stricture before intervention. **(B)** The 0.035-inch guide wire was passed through the anastomosis. **(C)** Fluoroscopic view shows that the guide wire is visible into the gastric cavity and the displaced metal stent is visible in the gastric cavity. **(D)** The stenosis was dilated with a biliary dilator along the guide wire. **(E)** The stenosis was dilated to a diameter of 10 mm using a biliary balloon. **(F)** Endoscopic view of the esophageal anastomosis stricture after intervention.

An endoscopic examination was performed using a 6 mm gastroscope under propofol sedation. The esophagogastric anastomosis was located 24 cm from the incisors, with the mucosa appearing smooth. Notably, due to scar constriction, the anastomosis was narrow and nearly occluded, making it challenging to pass even the finest guide wire (Figure 1A). A 0.035-inch wire guide (No. AG-5041-3545) was utilized. The “sliding” end is introduced through the biopsy channel of the upper endoscope and gently moved to pass through the narrow area of the anastomosis, using a technique like inserting a guide wire into the bile duct (Figure 1B).

Under both endoscopic and X-ray monitoring, the wire guide successfully penetrated the stenosis and entered the stomach (Figure. 1C). Subsequently, a papillary sphincterotomy knife (Model No. KD-V411M-0725) was advanced into the stomach along the guide wire. Contrast medium was injected to confirm the position of the cannula in the stomach under fluoroscopy. Following this, 8.5 F and 10 F biliary dilators were positioned along the guide wire for dilation and maintained for 2 min (Figure. 1D). A slight resistance was noted during the passage of the biliary dilator. Afterward, the esophagus was visualized with contrast to confirm the guide wire’s position, and the stenosis was dilated to a diameter of 10 mm using a balloon for 3 min, until the peak was eliminated (Figure 1E). Post-dilation, a minor amount of oozing blood was observed at the anastomosis site (Figure 1F).

**Figure 2:**
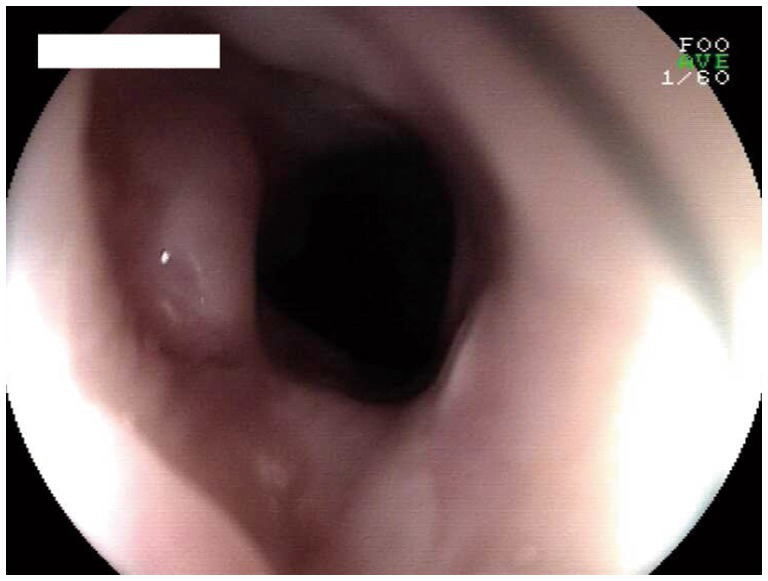
Endoscopic view of the anastomosis at 10 weeks after treatment.

Over the subsequent 6 weeks, dilation treatment was performed six times utilizing a Savary-Gilliard dilator (No. JHK15061301). Ultimately, endoscopy demonstrated that the esophageal stricture had significantly improved, allowing for the easy passage of a 9.8 mm endoscope (Figure 2), and the patient’s eating obstruction symptoms were completely resolved. There was no recurrence of stenosis noted upon endoscopy 4 weeks later.

## Discussion

Esophageal strictures, particularly in patients with a history of esophagectomy and subsequent radiotherapy, present significant management challenges. Traditional therapeutic approaches, including endoscopic dilation, stent placement, and pharmacological treatments, frequently result in suboptimal outcomes, characterized by high recurrence rates and the potential for adverse complications. This case report explores the application of biliary accessories in the endoscopic management of complete EAS, highlighting the potential of this approach for managing complex cases.

The current literature emphasizes the increasing prevalence of dysphagia in post-esophagectomy patients, often due to complications such as anastomotic stricture and scarring from radiation therapy (2). Research indicates that esophageal strictures can be effectively treated using various endoscopic techniques, including endoscopic dilation, endoscopic incision, stent placement, and pharmacological therapy (1). Studies have shown that endoscopic bougie dilation is comparable to fluoroscopic balloon dilation in terms of safety and efficacy; however, bougie dilation may have a higher recurrence rate due to diameter limitations compared to balloon dilation (3). Endoscopic incision, which alleviates symptoms by radially or circumferentially incising the stricture scar, has been proven to be safe and effective (4). Stent placement serves as a temporary measure that can rapidly ameliorate dysphagia in patients, although its long-term efficacy and risk of complications warrant further evaluation (5). Pharmacological therapy, including the concurrent local injection of corticosteroids during dilation, aims to reduce scar formation and enhance the effectiveness of dilation (6–8). The choice of these therapeutic methods and their outcomes can vary among individuals.

Safe and effective dilation therapy is essential to relieve patient suffering and improve prognosis. For some patients with refractory EAS, it is difficult for the guide wire to pass through the stenosis segment, and blind insertion can easily cause tissue damage, bleeding, and even perforation (9). The current landscape of endoscopic treatment for refractory EAS highlights the limitations of traditional methods like Savary-Gilliard’s bougie dilation, which often yield suboptimal short-term and long-term outcomes (10). Combined antegrade retrograde dilation offers significant advantages in the management of EAS, including enhanced success rates, reduced complications, and greater flexibility. However, the technique also presents challenges such as technical complexity, longer procedural times, and higher costs (3). Endoscopic radial incision has shown superior efficacy and safety, with significantly higher effective rates and fewer complications, making it a promising alternative for managing refractory EAS (4, 11). Longitudinal incisions in the esophageal muscle can effectively reduce pressure at the stenotic site and restore patency of the esophagus. However, in the present case, the patient’s cardiovascular condition added an additional layer of risk. Furthermore, the presence of a metal stent and the complexity of the gastric anatomy posed significant challenges, necessitating an alternative approach.

In this case, a 66-year-old man with a history of esophageal cancer and radiotherapy presented with severe dysphagia due to EAS. Despite multiple attempts at dilation and stent placement, the stricture persisted and nearly completed, necessitating a novel approach. Biliary intubation and biliary dilation, originally designed to dilate biliary obstruction and stenosis, can navigate through narrow and complex anatomical structures, making them suitable for patients with distorted anatomy due to prior treatments. Previously, Ahlawat et al. (12) successfully used biliary accessories for antegrade dilation of complex upper esophageal strictures in head and neck cancer patients, significantly improving dysphagia and achieving long-term patency.

The use of biliary accessories, including a fine guide wire, papillary sphincterotomy, and biliary dilators, was employed to achieve successful dilation. This method minimizes the risk of complications such as perforation and bleeding, which are common with blind insertion of guide wires. Studies have shown that biliary accessory dilation can result in lower recurrence rates compared to traditional methods. However, there are also disadvantages to consider. The technique requires specialized skills and equipment, which may not be readily available in all clinical settings. The economic aspects, including the cost of biliary accessories and the potential for increased procedural time, should be considered.

Further research is warranted to validate the efficacy and broader applicability of this innovative technique in similar clinical scenarios. Technological advancements, such as more advanced imaging modalities and endoscopic ultrasound-guided precise dilation tools, could further improve the technique. Developing clinical guidelines to standardize the use of biliary accessories in the treatment of esophageal strictures is also essential.

## Data Availability

The original contributions presented in the study are included in the article/supplementary material, further inquiries can be directed to the corresponding author.

## References

[1] Canakis A Kesar V Twery B Ali O Canakis J Hudspath C. The efficacy and safety of treatment outcomes for refractory benign esophageal strictures using a novel combination of needle-knife stricturoplasty, balloon dilation, and steroid injection (with video). GE Port J Gastroenterol. (2024) 31:48–53. doi: 10.1159/000527770, PMID: 38476305 PMC10928867

[2] Edmondson J Hunter J Bakis G O’Connor A Wood S Qureshi AP. Understanding post-esophagectomy complications and their management: the early complications. J Clin Med. (2023) 12:7622. doi: 10.3390/jcm12247622, PMID: 38137691 PMC10743498

[3] Fukushi G Fujimoto A Hara K Nishikawa Y Matsuno T Matsuda T. Fluoroscopic balloon dilatation with antegrade and retrograde endoscopes is useful for complete pharyngoesophageal obstruction after radiation therapy. Endoscopy. (2022) 54:E931–2. doi: 10.1055/a-1858-4558, PMID: 35790182 PMC9736824

[4] Ma X Zhang X Li B Zhu T Ma T Zhang X. Endoscopic stricturotomy in the treatment of refractory esophageal anastomotic strictures. Dysphagia. (2023) 38:650–6. doi: 10.1007/s00455-022-10495-5, PMID: 35859043

[5] Bi Y Li J Yi M Yu Z Han X Ren J. Self-expanding segmental radioactive metal stents for palliation of malignant esophageal strictures. Acta Radiol. (2020) 61:921–6. doi: 10.1177/0284185119886315, PMID: 31744304

[6] Sakaguchi Y Tsuji Y Shinozaki T Ohki D Mizutani H Minatsuki C. Steroid injection and polyglycolic acid shielding to prevent stricture after esophageal endoscopic submucosal dissection: a retrospective comparative analysis (with video). Gastrointest Endosc. (2020) 92:1176–1186.e1. doi: 10.1016/j.gie.2020.04.070, PMID: 32376336

[7] Tanaka K Kikutani T Tamura F Sato S Komagata Y Shibasaki I. Problems experienced when swallowing solid oral dosage forms in older Japanese patients with dysphagia: a cross-sectional study. Spec Care Dentist. (2024) 44:214–20. doi: 10.1111/scd.12853, PMID: 37029091

[8] Teng L Yang X Ding J. Comparison of the prevention of esophageal stricture between oral prednisolone alone and oral prednisolone combined with nasogastric tube in superficial esophageal cancer after endoscopic submucosal dissection. Turk J Gastroenterol. (2024) 35:481–7. doi: 10.5152/tjg.2024.23487, PMID: 39128118 PMC11232071

[9] Ren LH Ge M Ding J Zhu H Li YY Lu Q. Safety and efficacy of yellow zebra guide wire exchange system in the treatment of complete upper digestive stenosis. Zhonghua Yi Xue Za Zhi. (2023) 103:3133–5. doi: 10.3760/cma.j.cn112137-20230531-00899, PMID: 37840185

[10] Lu J Pan R Fu J Li S Ji R Lu X. Benefits of precise endoscopic incision on post-dilation mucosal scars to treat refractory esophageal stricture after endoscopic submucosal dissection. Endosc Int Open. (2023) 11:E409–12. doi: 10.1055/a-2048-1532, PMID: 37102186 PMC10125776

[11] Wang F Zhang D Zeng J Chen J. Comparison of endoscopic radial incision and Savary-Gilliard’s bougie dilation in efficacy on refractory esophagogastric anastomosis strictures. Ann Palliat Med. (2021) 10:10963–70. doi: 10.21037/apm-21-2648, PMID: 34763459

[12] Ahlawat SK Davidson BJ Al-Kawas FH. Successful use of biliary accessories in antegrade dilation of complex upper esophageal stricture due to chemoradiation and surgery. Dis Esophagus. (2008) 21:86–9. doi: 10.1111/j.1442-2050.2007.00705.x, PMID: 18197945

